# Peripheral cytokine interleukin‐10 alleviates perihematomal edema after intracerebral hemorrhage via interleukin‐10 receptor/JAK1/STAT3 signaling

**DOI:** 10.1111/cns.14796

**Published:** 2024-06-12

**Authors:** Yao Xu, Kaishan Wang, Yalan Dai, Wei Yang, Xufang Ru, Wenyan Li, Hua Feng, Gang Zhu, Qin Hu, Yujie Chen

**Affiliations:** ^1^ Department of Neurosurgery and State Key Laboratory of Trauma and Chemical Poisoning, Southwest Hospital Third Military Medical University (Army Medical University) Chongqing China; ^2^ Chongqing Key Laboratory of Intelligent Diagnosis, Treatment and Rehabilitation of Central Nervous System Injuries, Southwest Hospital Third Military Medical University (Army Medical University) Chongqing China; ^3^ Chongqing Clinical Research Center for Neurosurgery, Southwest Hospital Third Military Medical University (Army Medical University) Chongqing China; ^4^ Department of Neurosurgery, Ren Ji Hospital Shanghai Jiao Tong University School of Medicine Shanghai China

**Keywords:** blood–brain barrier, Interleukin‐10, intracerebral hemorrhage, perihematomal edema, tight junction

## Abstract

**Aims:**

The extent of perihematomal edema following intracerebral hemorrhage (ICH) significantly impacts patient prognosis, and disruption of the blood–brain barrier (BBB) exacerbates perihematomal edema. However, the role of peripheral IL‐10 in mitigating BBB disruption through pathways that link peripheral and central nervous system signals remains poorly understood.

**Methods:**

Recombinant IL‐10 was administered to ICH model mice via caudal vein injection, an IL‐10‐inhibiting adeno‐associated virus and an IL‐10 receptor knockout plasmid were delivered intraventricularly, and neurobehavioral deficits, perihematomal edema, BBB disruption, and the expression of JAK1 and STAT3 were evaluated.

**Results:**

Our study demonstrated that the peripheral cytokine IL‐10 mitigated BBB breakdown, perihematomal edema, and neurobehavioral deficits after ICH and that IL‐10 deficiency reversed these effects, likely through the IL‐10R/JAK1/STAT3 signaling pathway.

**Conclusions:**

Peripheral IL‐10 has the potential to reduce BBB damage and perihematomal edema following ICH and improve patient prognosis.

## Graphical abstract

The diagram shows that after intracranial hemorrhage, peripherally cytokine IL‐10 binds to the IL‐10 receptor on endothelial cells, induces the expression of tight junction proteins via JAK1/STAT3 signaling, and alleviates blood–brain barrier damage. IL‐10 receptor knockdown can partially reverse this effect by specifically inhibiting the expression of the IL‐10 receptor.

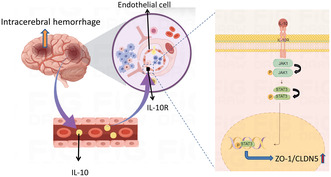

## 1 INTRODUCTION

Hypertensive intracerebral hemorrhage (ICH) is a common and often fatal injury to the central nervous system (CNS) that frequently occurs within the basal ganglia. Surviving patients experience various irreversible functional impairments and sequelae.^1^ In addition to the initial anatomical damage caused by ICH, secondary damage resulting from the subsequent inflammatory response is common, leading to blood–brain barrier (BBB) disruption and exacerbation of cerebral edema.^2^ Perihematomal edema frequently occurs shortly after ICH and its rapid growth may lead to severe intracranial hypertension as large as the initial hematoma. Inflammation‐mediated BBB injury is considered to be the primary cause of perihematomal edema.^3^ Therefore, the prevention of BBB disruption and perihematomal edema is a key focus in ICH treatment.

Unlike other cytokines whose expression significantly changes after ICH, interleukin‐10 (IL‐10) is a critical anti‐inflammatory cytokine that inhibits proinflammatory signaling and immune responses after ICH. A previous study showed that IL‐10‐deficient mice exhibited more severe neuroinflammation, brain edema, iron deposition, and neurological deficits after ICH, which were primarily associated with delayed hematoma clearance.^4^ IL‐10 primarily signals via signal transducer and activator of transcription 3 (STAT3),^5^ a transcription factor that is phosphorylated by Janus tyrosine protein kinase 1 (JAK1) upon IL‐10 receptor activation. This process leads to STAT3 dimerization, nuclear translocation, and subsequent transcriptional activation of anti‐inflammatory genes,^6^ potentially reducing neuroinflammation and BBB damage while improving neurological function.

Although a previous study reported that ICH triggers IL‐10 release in the blood,^7^ whether peripheral IL‐10 rather than IL‐10 in the brain parenchyma affects BBB disruption and perihematomal edema after ICH has not been thoroughly investigated. Therefore, the aim of the present study was to investigate whether peripherally derived IL‐10 mitigates BBB breakdown, subsequently alleviating perihematomal edema and neurobehavioral dysfunction in an ICH mouse model, with a specific focus on JAK1/STAT3 signaling‐mediated BBB protection.

## 2 MATERIALS AND METHODS

### 2.1 Experimental animals

All the experimental procedures conducted in this study were rigorously reviewed and approved by the Laboratory Animal Welfare and Ethics Committee of the Third Military Medical University (AMUWEC20232224). Adherence to ethical standards and guidelines was paramount, and all protocols were meticulously executed in accordance with the principles outlined in the National Institutes of Health Guide for the Care and Use of Laboratory Animals. Furthermore, the results are reported in accordance with the Animal Research: Reporting of In Vivo Experiments, Version 2.0 (ARRIVE 2.0) guidelines, ensuring comprehensive and transparent documentation of our methodologies. A total of 238 male C57BL/6N mice aged 8–12 weeks and weighing between 24 and 28 g were acquired from the Animal Experimental Center of the Third Military Medical University, located in Chongqing, China. Throughout the duration of the study, stringent measures were implemented to maintain optimal housing conditions for the animals, including a specific pathogen‐free environment. The temperature was maintained in the range of 23–25°C, the animals were housed on a consistent 12‐h light/dark cycle with ad libitum access to standard rodent chow and tap water. This meticulous attention to housing conditions allowed us to ensure the well‐being and physiological stability of the animal subjects, thereby maintaining the integrity and reliability of our experimental outcomes.

### 2.2 Experimental design

The experiment involved three distinct parts, as shown in Figure S1. The requisite number of animals for each part of the experiment, along with the corresponding mortality rates, are shown in Table S1. All experimental procedures and subsequent data analyses were performed in a double‐blind manner.

Experiment I: Assessment of IL‐10 levels after ICH. Thirty mice were randomly divided into five groups: the sham, ICH‐6 h, ICH‐1 d, ICH‐3 d, and ICH‐7 d groups. IL‐10 levels in both peripheral blood and the vicinity of the hematoma were measured via enzyme‐linked immunosorbent assay (ELISA) at the indicated time intervals (*n* = 6).
Experiment II: Evaluation of the impact of peripheral IL‐10 on BBB integrity, perihematomal edema and neurobehavioral function after ICH. Behavioral assessments (*n* = 6), an Evans blue (EB) extravasation assay (*n* = 6), and EB fluorescence analysis (*n* = 2) were conducted, and the brain water content was determined (*n* = 6). A total of 178 mice were randomly divided into four groups: the sham, ICH, ICH + recombinant mouse IL‐10 (ICH + rmIL10), and ICH + AAV2/9‐m‐IL‐10 (ICH + AAV‐IL‐10) groups. For all groups except the sham group, analyses were conducted at 1, 3, and 7 days post‐ICH. Additionally, eight mice from each group were subjected to double immunofluorescence staining (*n* = 2) and Western blotting (*n* = 6) 3 days post‐ICH to assess the expression of tight junction proteins, namely, ZO‐1 and CLDN5.
Experiment III: Elucidation of the effect of peripheral IL‐10 via the IL‐10R/JAK1/STAT3 signaling pathway after ICH. Mice were intraventricularly injected with either an IL‐10 knockout or control plasmid prior to ICH induction. An additional 28 mice were randomly divided into two groups: the ICH + rmIL‐10 + IL‐10R CRISP‐Cas9 knockout plasmid (ICH + rmIL‐10 + IL‐10R Cas9‐Plasmid) group and the ICH + IL‐10 + Control plasmid (ICH + rmIL‐10 + IL‐10R Ctr‐Plasmid) group. Western blotting (*n* = 6) was used to analyze the expression of p‐JAK and p‐STAT3 in each group 3 days post‐ICH. Furthermore, behavioral tests (*n* = 6) and EB extravasation assays (*n* = 6), along with EB fluorescence analysis (*n* = 2), were conducted 3 days post‐ICH.

### 2.3 ICH model establishment

The experimental procedure was performed as previously described.^8^ In brief, the mice were anesthetized with isoflurane (RWD Life Sciences Ltd., China) and securely positioned in a stereotaxic apparatus (RWD Life Sciences Ltd., China). The cranial region was meticulously disinfected with iodophor, and then a midline incision was made to expose bregma. Utilizing bregma as a point of reference, the internal capsule was precisely localized employing a stereotaxic apparatus, with coordinates set at 0.8 mm anterior to bregma and 2.0 mm lateral to the midline. A small aperture, approximately 1 mm in diameter, was carefully made in the skull utilizing a high‐speed cranial drill. A total of 25 μL of autologous blood was administered with a sterile Hamilton syringe (catalog no. RN1702) coupled with a microinfusion pump (KD Scientific, Holliston, MA, USA). into the right basal ganglia of the mice at a depth of 3 mm from the dura mater at a controlled rate of 2 μL/min. After the injection, the needle was left in position for 5 min before being gradually withdrawn. A sham procedure in which mice were subjected to the same anesthetic regimen and immobilized on a stereotaxic instrument was performed. Following disinfection of the dermal surface and localization of the internal capsule, a similar cranial orifice was generated. After syringe removal, the aperture in the skull was sealed utilizing bone wax, and the surgical site was disinfected and sutured. The mice were then placed on a warming pad until they regained consciousness. These procedures were carried out under stringent aseptic conditions within an ultraclean laboratory, which was sanitized via ultraviolet light exposure.

### 2.4 Drug administration

Recombinant mouse IL‐10 protein, procured from Yeasen Biotechnology (Shanghai), was reconstituted in phosphate‐buffered saline (PBS). Subsequently, it was injected into the experimental mice at a dose of 0.3 μg via the caudal vein precisely 1 h after the induction of ICH.^9^ For the specific silencing of IL‐10 expression, an IL‐10‐inhibiting virus (2 μL/mouse; HBAAV2/9‐m‐IL‐10 shRNA2‐EGFP; Hanheng Biotechnology, Shanghai, China) was intracerebroventricularly injected. Conversely, to inhibit IL‐10 receptor (IL‐10R) expression, the mice were intracerebroventricularly injected with an IL‐10R CRISPR‐Cas9 knockout plasmid (2 μL/mouse; catalog no. PPL50755; Geneppl Technology, Nanjing, China) or a control plasmid (2 μL/mouse).

### 2.5 Intraventricular injection

Intraventricular injections were administered as previously described.^10^ Mice were subjected to deep anesthesia using isoflurane (R510‐22; RWD Life Sciences Ltd., China) and subsequently secured onto a stereotaxic apparatus (RWD Life Sciences Ltd., China). A small burr hole was created in the skull using a high‐speed cranial drill. The specific location of the hole was 0.22 mm posterior to bregma and 1.0 mm lateral to the midline. The IL‐10‐inhibiting virus or IL‐10 knockout plasmid was delivered to the right ventricle at a steady rate of 0.67 μL/min. Following an 8‐min interval, the syringe was slowly withdrawn and the burr hole was meticulously sealed with bone wax.

### 2.6 Basso Mouse Scale (BMS)

The BMS was used to assess the motor function of the mice. BMS scores range from 0, denoting the absence of ankle movement, to 9, indicating frequent or consistent plantar stepping characterized by predominantly coordinated actions, parallel orientation of the paws during initial contact and lifting, maintenance of normal trunk stability, and a consistently elevated tail.^11^

### 2.7 Open field test (OFT)

Exploratory activity was assessed utilizing the OFT.^12^ The open field apparatus was partitioned uniformly into four squares, each measuring 50 cm × 50 cm × 40 cm. Prior to experimentation, the mice were acclimatized to the environment for 1 h, and pertinent parameters were determined using Viewpoint software (Consent Biotechnology Co., Ltd., Changsha, China). Subsequently, each mouse was positioned at the center of the open field apparatus facing the same direction and observed for 5 min. The locomotor activity and trajectories of the mice were captured using a video imaging system (Consent Biotechnology Co., Ltd., Changsha, China). The average speed was computed based on the distance traversed by the mouse during the observation period.

All behavioral assessments were randomly allocated, and the researcher conducting the analyses was blinded to the specific drugs administered.

### 2.8 Brain water content (BWC)

The BWC was assessed at three time points (1, 3, and 7 days post‐ICH) in accordance with the established literature. The mice were subjected to deep anesthesia using isoflurane (R510‐22; RWD Life Sciences Ltd., China) and subsequently euthanized, after which their brains were promptly excised. After removal, the brains were divided into five distinct regions: the basal ganglia, cortex, cerebellum, hippocampus, and brainstem. Each brain region was weighed using an analytical microbalance to determine the wet weight. The brain tissue was then desiccated in an oven at 105°C for a period of 72 h and subsequently reweighed to determine the dry weight. The percentage of water content was computed utilizing the formula [(wet weight – dry weight)/wet weight] × 100%.^13^

### 2.9 EB extravasation and fluorescence analysis

The EB assay was conducted in accordance with established protocols.^13^ Briefly, the mice were immobilized in a tail vein injection device and administered a 2% EB dye solution (5 mL/kg; E2129; Sigma–Aldrich) via the tail vein. Following injection, the needle was kept in place for 1 min to prevent reflux. Subsequently, the dye was allowed to circulate for 2 h, after which the mice were anesthetized using isoflurane (R510‐22; RWD Life Sciences Ltd., China) and orally administered cold PBS. The brain tissue was then promptly harvested.

The right cerebral hemisphere was weighed, and normal saline (NS; 400 μL) was added for subsequent homogenization. The samples were centrifuged at 15,000 *g* for 30 min. An equivalent volume of 50% trichloroacetic acid was added to the resulting supernatant, followed by overnight incubation at 4°C and subsequent centrifugation at 15,000 *g* for 30 min. The absorbance of the EB dye was measured spectrophotometrically (at 615 nm; Varioskanfla, Thermo Fisher Scientific). The EB content was calculated utilizing a standard curve, and the results are expressed as μg of EB dye per gram of brain tissue. For EB fluorescence analysis, brains were collected immediately after NS administration and transcardially perfused with 4% paraformaldehyde. Frozen coronal brain sections (20 μm) were prepared and stained with 4′,6‐diamidino‐2‐phenylindole (DAPI). Subsequently, confocal laser scanning microscopy (mRFP 555; ZISS 880, Carl Zeiss Microscopy Ltd., England) was employed to observe EB fluorescence.

### 2.10 Immunofluorescence staining

Immunofluorescence staining of cryosections from frozen brain specimens was conducted in accordance with previously reported procedures.^14^ Specifically, 3 days post‐ICH, the mice were subjected to deep anesthesia followed by intravenous administration of NS and 4% paraformaldehyde. The brains were promptly excised, fixed in 4% paraformaldehyde for 24 h, and then immersed in 30% sucrose for 72 h for cryoprotection. Serial coronal sections (20 μm thick) were obtained using a freezing microtome (CM1860UV; Leica, Wetzlar, Germany) and permeabilized with 0.5% Triton X‐100 in phosphate‐buffered saline (PBS) for 30 min. The sections were blocked with 5% bovine serum albumin for 2 h and incubated overnight at 4°C with the following primary antibodies: rabbit polyclonal anti‐ZO1 (diluted 1:200; PB9234; BOSTER), rabbit polyclonal anti‐CLDN5 (diluted 1:200; 34‐1600; Thermo Fisher Scientific), and mouse monoclonal anti‐CD31 (diluted 1:200; 14‐0311‐82; Thermo Fisher Scientific) antibodies. Following thorough washing with PBS, the sections were incubated with fluorescent secondary antibodies for 2 h at room temperature. The secondary antibodies utilized were Alexa Fluor 488‐ and Alexa Fluor 555‐conjugated secondary antibodies targeting mouse and rabbit immunoglobulins, respectively (dilution 1:500; Abcam). After staining with DAPI, the sections were subjected to confocal laser scanning microscopy (mRFP 555; ZISS 880, Carl Zeiss Microscopy Ltd., England) for visualization of fluorescence signals and image capture. Analysis of four distinct visual fields of the perihematomal region of each section was performed, with a total of three sections analyzed per mouse.

### 2.11 Western blot analysis

Western blot analysis was conducted in accordance with previously established protocols.^8^, ^13^, ^14^ Tissue samples surrounding the hematoma were harvested and homogenized, and a protein extraction kit (BC3710; Solarbio; Beijing, China) was used to extract total protein. Throughout the procedure, the temperature was strictly controlled to preserve protein integrity. Following sample preparation, equal amounts of sample protein (50 μg) were loaded onto SDS–PAGE gels. After the proteins were subjected to gel electrophoresis, they were transferred onto polyvinylidene fluoride (PVDF) membranes. The membranes were then blocked with 5% bovine serum albumin at room temperature for 2 h and subsequently incubated overnight at 4°C with primary antibodies, including rabbit polyclonal anti‐ZO1 (diluted 1:200; PB9234; BOSTER), rabbit polyclonal anti‐CLDN5 (diluted 1:200; 34–1600; Thermo Fisher Scientific), rabbit monoclonal anti‐STAT3 (diluted 1:1000; ab68153; Abcam), rabbit monoclonal anti‐pSTAT3 (diluted 1:1000; PA5‐17876; Thermo Fisher Scientific), rabbit monoclonal anti‐JAK1 (diluted 1:1000; ab133666; Abcam), rabbit polyclonal anti‐pJAK1 (diluted 1:1000; 44422G; Thermo Fisher Scientific), rabbit polyclonal anti‐IL‐10R (diluted 1:1000; 38,306; SAB), and mouse monoclonal anti‐glyceraldehyde‐3‐phosphate dehydrogenase (GAPDH) (diluted 1:5000; ab8245; Abcam). GAPDH served as an internal loading control. Following primary antibody incubation, the membranes were incubated with specific horseradish peroxidase‐conjugated secondary antibodies (1:10,000 dilution; Abcam) for 2 h at room temperature. Immunoreactive bands were visualized and detected using an enhanced chemiluminescence reagent kit (Thermo Scientific, Rockford, IL, USA) and a bioimaging system (ChemiDoc XRS+; Bio‐Rad, Hercules, CA, USA). The intensity of each band was quantified using Image Lab software (version 3.0; Bio‐Rad, Hercules, CA, USA).

### 2.12 ELISA

An ELISA kit (EMC00, NeoBioscience Biotech Ltd., Shenzhen, China) specific for mouse IL‐10 was used to quantify IL‐10 levels in brain tissue on the side of the cerebral hemorrhage and in serum specimens. All the experimental procedures were conducted in accordance with the protocols provided by the manufacturer.

### 2.13 Statistical analysis

Statistical analyses and data graphing were performed utilizing GraphPad Prism 8.4.2 software (GraphPad Software, USA). Continuous variables are presented as the mean ± standard deviation (SD), while discrete variables are presented as the median ± interquartile range (IQR). We used the Shapiro–Wilk normality test to assess the data distribution. All the data were subjected to tests for normality. For comparisons of more than two groups, one‐way analysis of variance (ANOVA) followed by appropriate post hoc tests was employed for normally distributed data, whereas nonnormally distributed data were subjected to nonparametric tests, specifically the Kruskal–Wallis test. Furthermore, two‐way repeated‐measures ANOVA was utilized to compare behavioral data across different time points and experimental groups. P < 0.05 was used as the threshold for statistical significance.

## 3 RESULTS

### 3.1 Mortality

In this study, the overall mortality rate among the mice was determined to be 3.36% (8 of 238), with no statistically significant variance observed in mortality rates across the experimental groups (Table S1). Furthermore, no mice were deemed ineligible for inclusion or excluded from the analysis.

### 3.2 Timeline of IL‐10 expression in brain and peripheral after ICH

**FIGURE 1:**
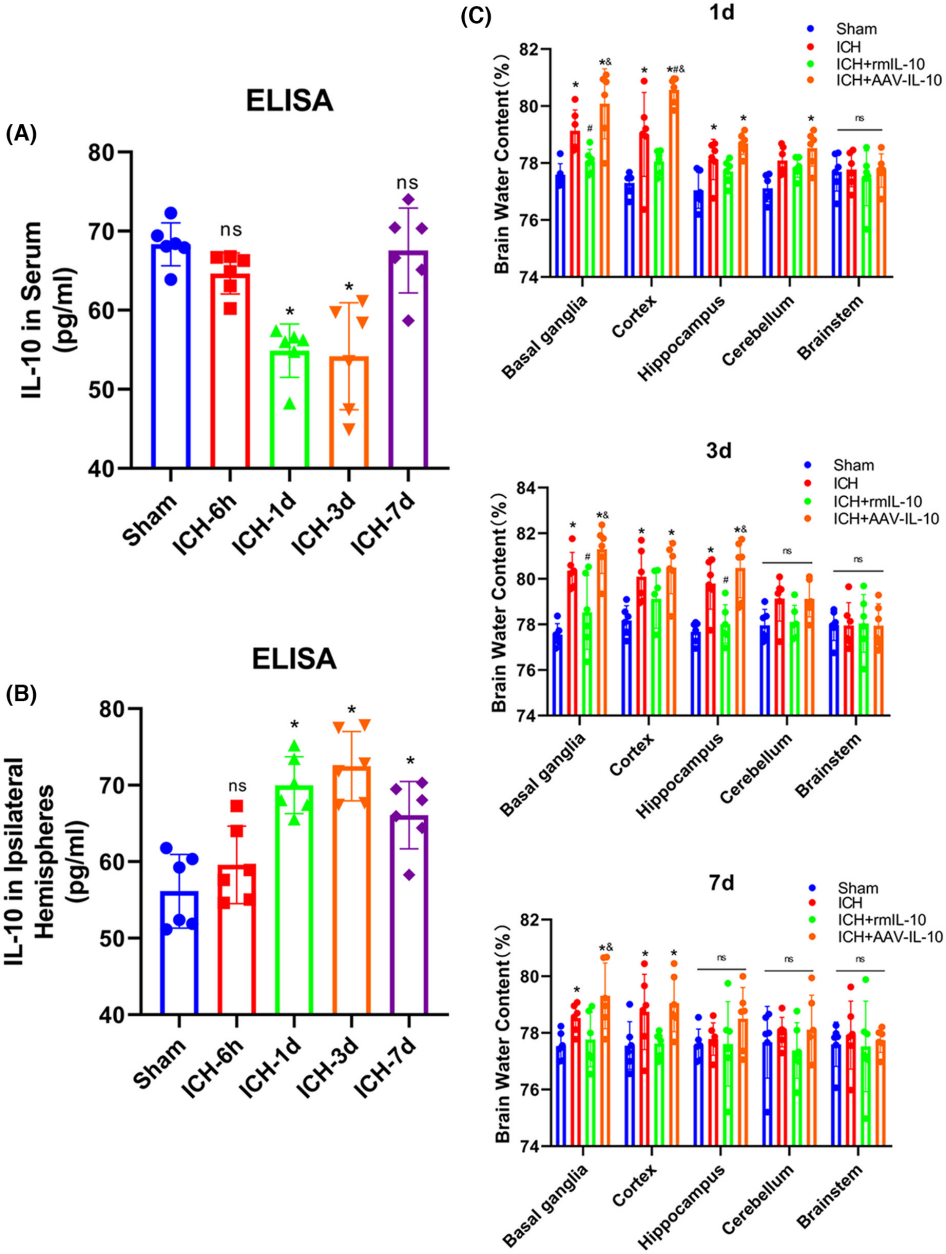
Changes in the expression of IL‐10 and the effect on perihematomal edema after ICH. (A) Quantitative analysis of the change in IL‐10 levels in the serum over time. (B) Quantitative analysis of the change in IL‐10 levels over time on the side of the cerebral hemorrhage. (C) Statistical analysis of brain water content in different brain regions on the side of cerebral hemorrhage, including the basal ganglia, cortex, cerebellum, hippocampus, and brainstem, at 1, 3, and 7 days after ICH. The data are expressed as the mean ± SD. Sham, sham‐operated group; ICH‐6 h, 6 h after ICH; ICH‐1 day, 1 day after ICH; ICH‐3 day, 3 day after ICH; ICH‐7 day, 7 day after ICH. *p < 0.05 versus the sham group; ^#^ *p* < 0.05 versus the ICH group; ^&^ *p* < 0.05 versus the ICH + rmIL‐10 group; ns: no significance.

The endogenous expression of IL‐10 in brain tissue adjacent to the hematoma as well as in peripheral blood was assessed using ELISA. Notably, the IL‐10 levels in peripheral blood decreased, reaching the lowest point at 3 days post‐ICH, followed by a gradual increase (Figure 1A). Conversely, IL‐10 expression in the brain tissue surrounding the hematoma exhibited a notable increase, peaking at 3 days post‐ICH (Figure 1B).

### 3.3 Peripheral IL‐10 alleviates perihematomal edema after ICH

Cerebral edema was evaluated through BWC analysis of various intracranial regions at 1, 3, and 7 days following ICH. Notably, at each time point assessed, a significant increase in BWC was detected in both the basal ganglia and cortex in the ICH group compared with the sham group (*p* < 0.05; Figure 1C). Moreover, edema in the hippocampal region was observed at 3 days post‐ICH (*p* < 0.05; Figure 1C). Furthermore, the absence of IL‐10 exacerbated the observed edematous response; conversely, peripheral IL‐10 significantly mitigated brain edema (*p* < 0.05; Figure 1C). Intriguingly, no significant alterations in BWC were evident in the cerebellum or brain stem across the experimental groups (*p* > 0.05; Figure 1C).

### 3.4 Peripheral IL‐10 mitigates BBB breakdown after ICH

**FIGURE 2:**
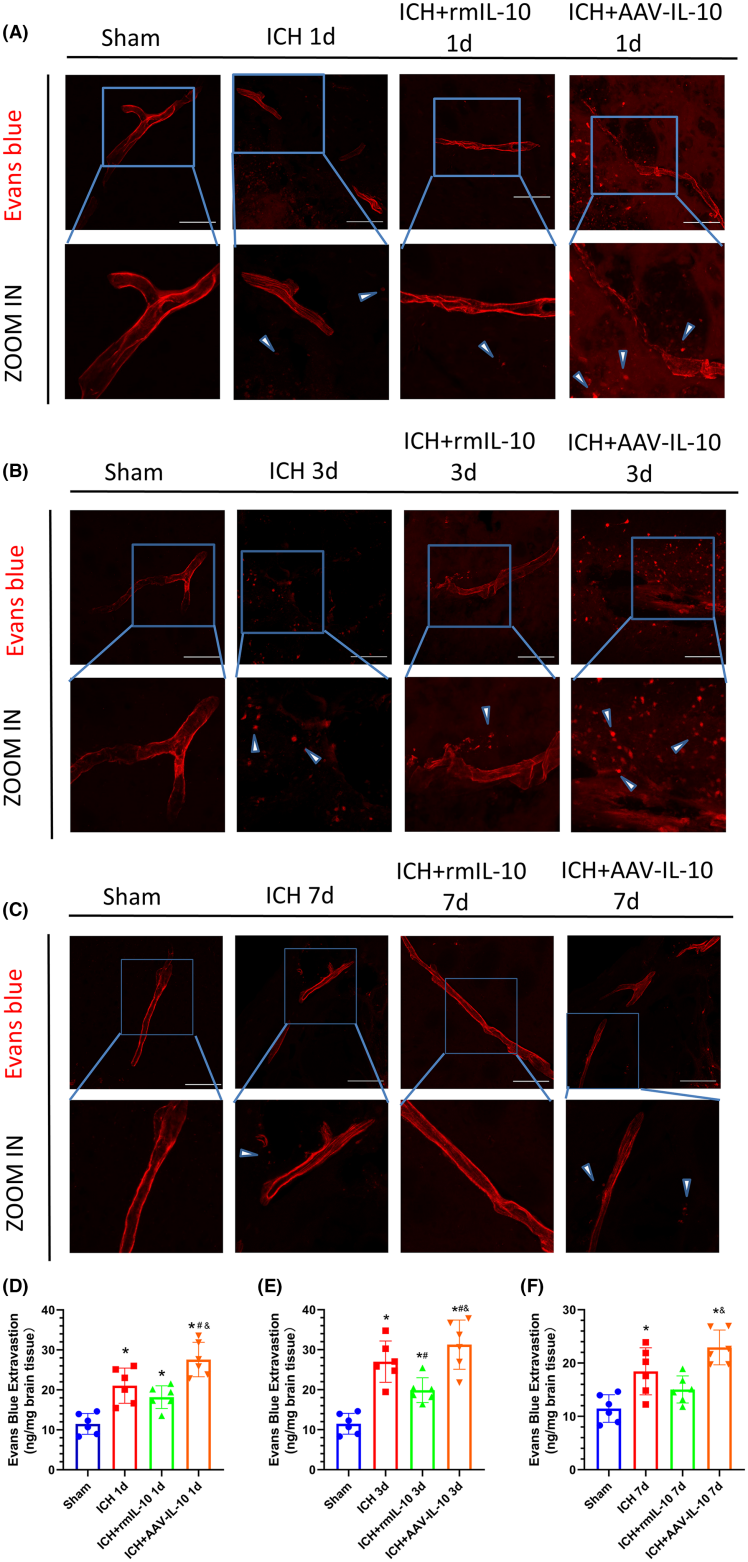
Peripheral IL‐10 alleviated BBB damage at 1, 3, and 7 days after ICH. (A–C) EB fluorescence images and magnified images; scale bar = 100 μm. The arrows in the magnified images indicate EB dye extravasated from the vessels. (D–F) Quantitative analysis of EB extravasation. The data are expressed as the mean ± SD. *p < 0.05 versus the sham group; ^#^ *p* < 0.05 versus the ICH group; ^&^ *p* < 0.05 versus the ICH + rmIL‐10 group; ns: no significance.

We conducted an EB extravasation assay to evaluate BBB integrity. Notably, EB extravasation was significantly greater in the ipsilateral (right) hemisphere on days 1, 3, and 7 following ICH than in the sham group (*p* < 0.05; Figure 2A–F). Furthermore, IL‐10 deficiency was associated with increased EB extravasation, while peripheral IL‐10 administration mitigated this effect (*p* < 0.05; Figure 2A–F). The most pronounced EB extravasation was observed at 3 days post‐ICH (Figure 2B, E), which coincided with the peak of cerebral edema, which was particularly evident in the basal ganglia, cortex, and hippocampus, as determined by BWC analysis (Figure 1C). Collectively these findings suggest that the 3‐day post‐ICH time point represents a critical point characterized by severe cerebral edema and BBB disruption; thus, we selected this time point as the primary time point for subsequent experiments.

**FIGURE 3:**
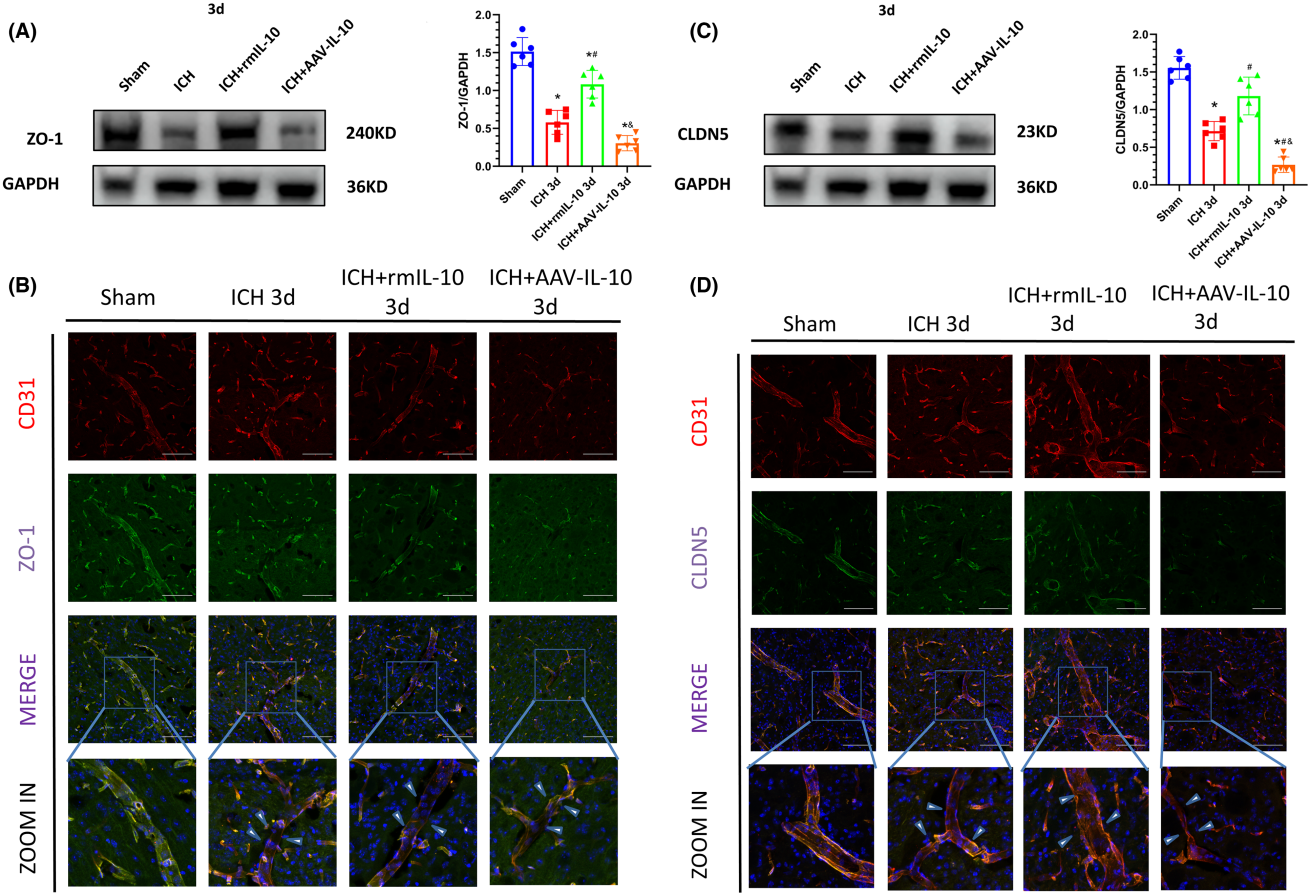
Peripheral IL‐10 upregulated the expression of the tight junction proteins ZO‐1 and CLDN5 3 days after ICH. (A) Western blot images and quantitative analysis of ZO‐1 expression. The data are expressed as the mean ± SD.*p < 0.05 versus the sham group; ^#^ *p* < 0.05 versus the ICH group; ^&^ *p* < 0.05 versus the ICH + rmIL‐10 group; ns, no significance. (B) Double immunofluorescence staining images and magnified representative pictures of ZO‐1 (green) staining in endothelial cells (CD31, red). (C) Western blot images and quantitative analysis of CLDN5 expression. The data are expressed as the mean ± SD. *p < 0.05 versus the sham group; ^#^ *p* < 0.05 versus the ICH group; ^&^ *p* < 0.05 versus the ICH + rmIL‐10 group; ns, no significance. (D) Double immunofluorescence staining images and magnified representative images of CLDN5 (green) staining in endothelial cells (CD31, red). Scale bar = 100 μm. The arrows in the magnified images indicate regions in which tight junction protein levels were significantly decreased in vessels.

At 3 days post‐ICH, IL‐10 deficiency significantly attenuated the expression of tight junction proteins in the ipsilateral (right) cerebral hemisphere, while peripherally derived IL‐10 reversed this effect (Figure 3A–D). Western blot analysis revealed a notable reduction in the expression of ZO‐1 and CLDN5 in the ipsilateral hemisphere in the ICH + AAV‐IL‐10 group compared to the ICH group, whereas the ICH + rmIL‐10 group exhibited increased expression of ZO‐1 and CLDN5 (*p* < 0.05; Figure 3A,C). These findings were further corroborated by double immunostaining for CD31 (an endothelial cell marker) and ZO‐1 or CLDN5 (Figure 3B,D).

### 3.5 Peripheral IL‐10 alleviates apoplectic dyskinesia after ICH, and IL‐10 deficiency has the opposite effect

**FIGURE 4:**
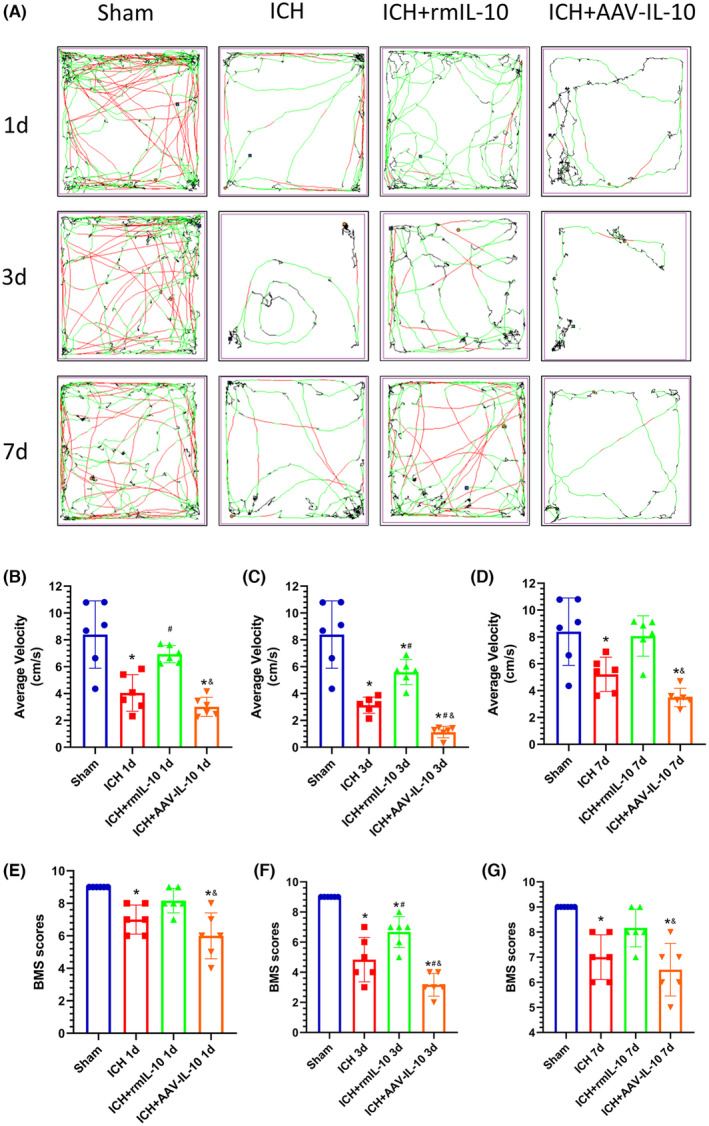
Peripheral IL‐10 alleviated apoplectic dyskinesia at 1, 3, and 7 days after ICH, and IL‐10 deficiency had the opposite effect. (A) Trajectories in the open field test and (B–D) statistical analysis of the average velocity of the mice. According to the preset parameters, the trajectories of the mice are presented in three colors: black (low speed), green (medium speed), and red (high speed). (E–G) Statistical analysis of the BMS scores of the mice. The data are expressed as the mean ± SD or median and range. *p < 0.05 versus the sham group; ^#^ *p* < 0.05 versus the ICH group; ^&^ *p* < 0.05 versus the ICH + rmIL‐10 group; ns: no significance.

Motor function was assessed using the BMS and OFT on days 1, 3, and 7 post‐ICH. Both the BMS and mean velocity were lower in the ICH group than in the sham‐operated group, and this difference was particularly evident at 3 days post‐ICH (Figure 4A–G). Furthermore, comparative analysis of the mean speed and BMS scores revealed a notable reduction in motor function among the mice in the ICH + IL‐10 group compared to those in the ICH group on days 1, 3, and 7 post‐ICH. Conversely, the motor function of the mice in the ICH + IL‐10 group improved over time (*p* < 0.05; Figure 4A–G).

### 3.6 IL‐10R knockdown reverses the neuroprotective effects of IL‐10 via JAK2/STAT3 signaling at 3 days after ICH

**FIGURE 5:**
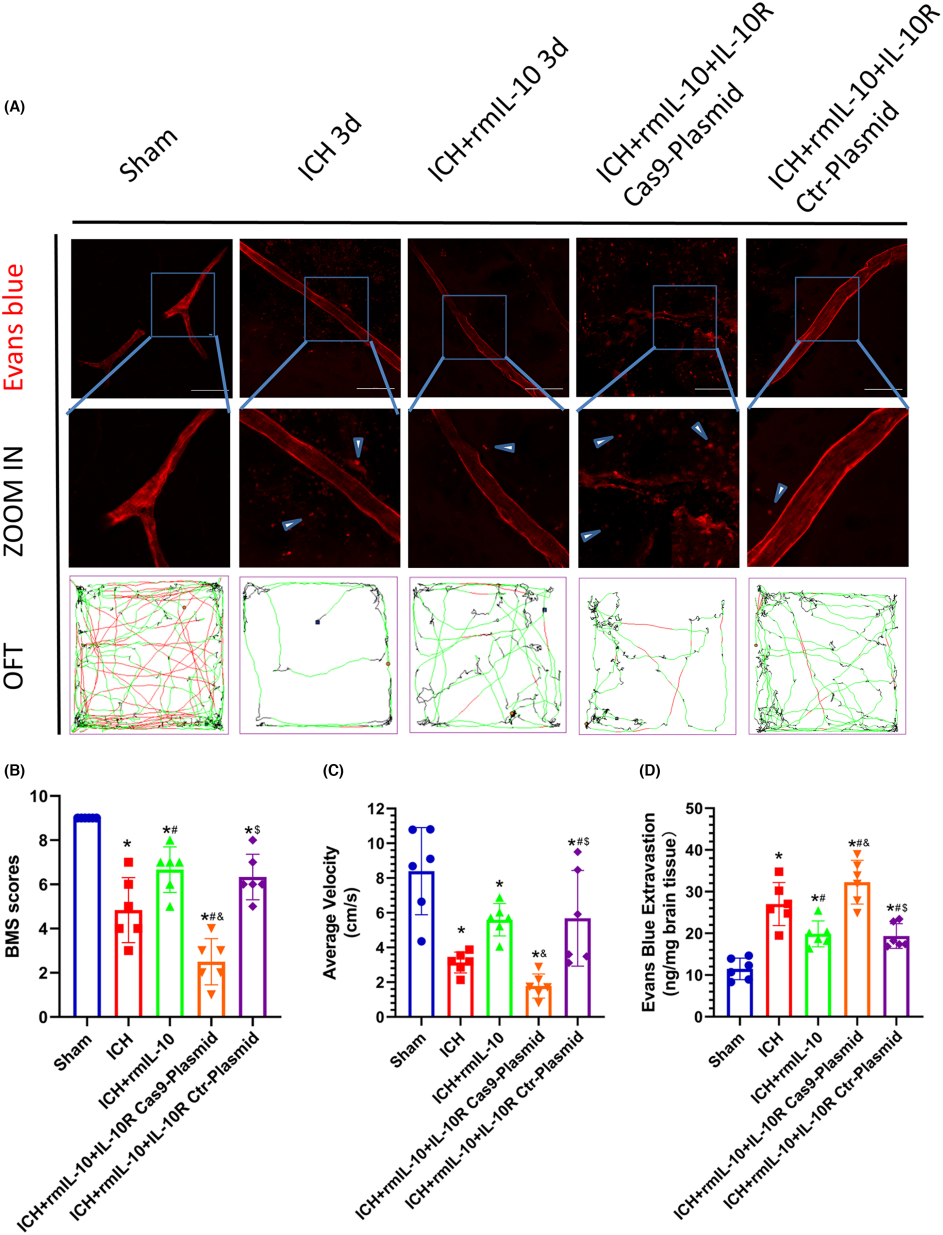
IL‐10R Cas9 plasmid‐mediated IL‐10R knockdown aggravated cerebral damage 3 days after ICH. (A) EB fluorescence images (scale bar = 100 μm) and trajectories in the OFT. According to the preset parameters, the trajectories of the mice are presented in three colors: black (low speed), green (medium speed), and red (high speed). (B–D) Statistical analysis of BMS scores, average velocity and EB extravasation. The data are expressed as the mean ± SD. *p < 0.05 versus the sham group; ^#^ *p* < 0.05 versus the ICH group; ^&^ *p* < 0.05 versus the ICH + rmIL‐10 group; ^&^ *p* < 0.05 versus the ICH + rmIL‐10 + IL‐10R Cas9‐Plasmid group; ns, no significance.

**FIGURE 6:**
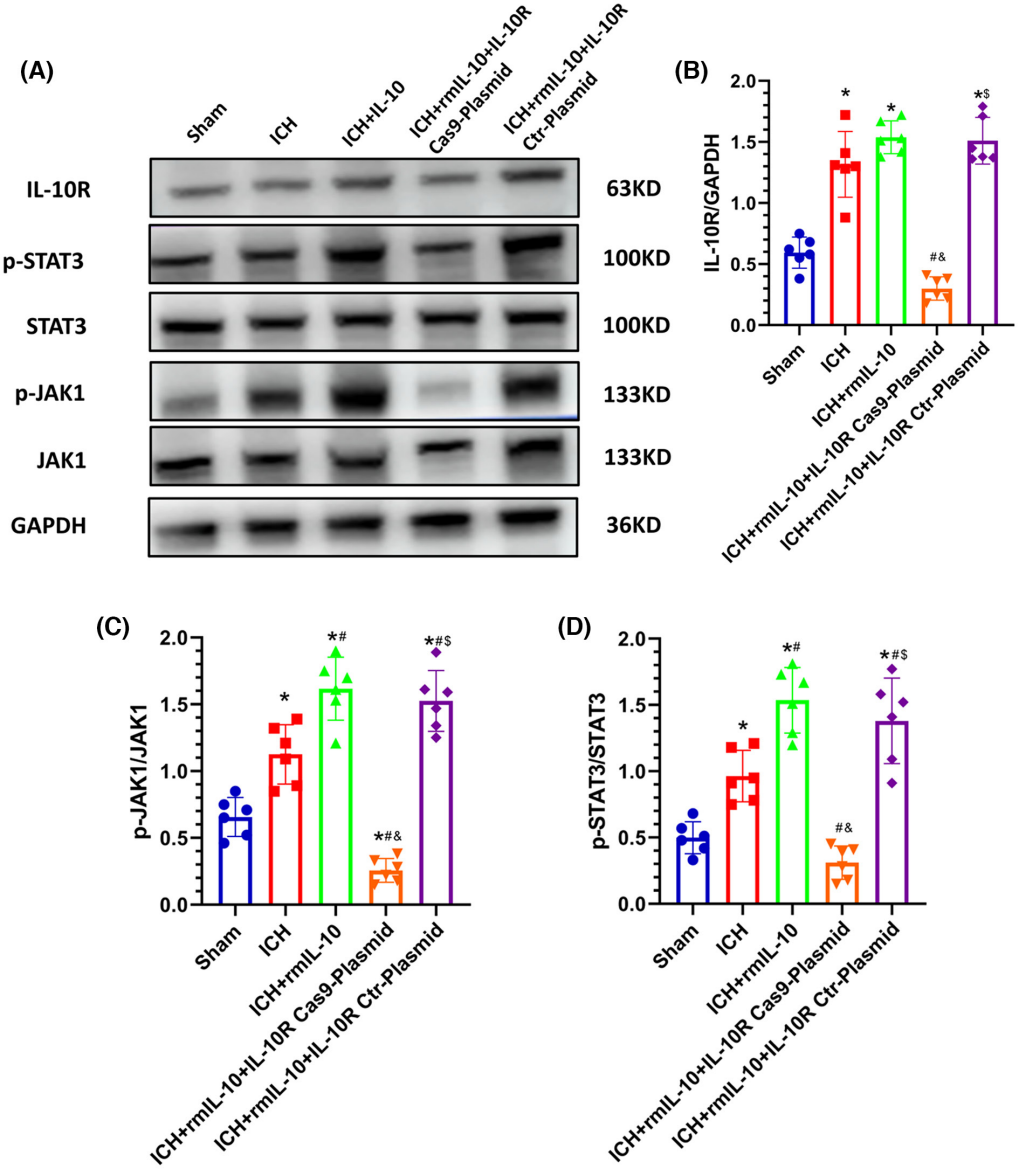
IL‐10R Cas9 plasmid‐mediated IL‐10R knockdown aggravated cerebral damage at 3 days after ICH via inhibition of the IL‐10R/JAK1/STAT3 signaling pathway. (A–D) Western blot images and quantitative analysis of IL‐10R, JAK1, p‐JAK1, STAT3, and p‐STAT3 levels on the side of cerebral hemorrhage in the sham, ICH, ICH + rmIL‐10, ICH + rmIL‐10 + IL‐10R Ctr‐Plasmid, and ICH + rmIL‐10 + IL‐10R Cas9‐Plasmid groups at 3 days after ICH.

These experimental findings elucidate the pivotal role of IL‐10R in mitigating intracerebral hemorrhage (ICH)‐induced BBB compromise and associated neurological deficits. Notably, pretreatment of mice with the IL‐10R Cas9 plasmid effectively downregulated IL‐10R expression, as corroborated by Western blot analysis (Figure 6A,B). BBB integrity and neurobehavioral function were evaluated via the EB extravasation test, BMS, and OFT. Remarkably, compared to those in the ICH‐alone group, the mice in the ICH group treated with IL‐10 exhibited a marked reduction in EB extravasation and significant increases in the BMS score and mean velocity (*p* < 0.05; Figure 4). However, the ICH + rmIL‐10 + IL‐10R Cas9 plasmid resulted in an increase in EB extravasation in the affected hemisphere, concomitant with a decrease in the mean velocity and BMS score (*p* < 0.05; Figure 5). These changes were not evident in the ICH + rmIL‐10 + IL‐10R Ctr plasmid group. Furthermore, the levels of downstream effectors of IL‐10R, namely, phosphorylated Janus kinase 1 (p‐JAK1) and phosphorylated signal transducer and activator of transcription 3 (p‐STAT3), were evaluated via Western blotting. Notably, a discernible reduction in the expression levels of p‐JAK1 and p‐STAT3 was observed in the ICH + rmIL‐10 + IL‐10R Ctr‐Plasmid group compared to the ICH + rmIL‐10 group (*p* < 0.05; Figure 6A,C,D), which supported the notion that the IL‐10R/JAK1/STAT3 signaling cascade mediates the salutary effects of peripherally administered IL‐10 on BBB preservation and motor function.

## 4 DISCUSSION

The findings of the present study indicate that IL‐10R inhibition exacerbates BBB disruption, perihematomal edema and neurological deficits after ICH, whereas exogenous IL‐10 administration ameliorates these deleterious consequences. Moreover, specific antagonism of IL‐10R via a plasmid exacerbates BBB compromise and motor dysfunction by attenuating IL‐10R/JAK1/STAT3 signaling pathway‐related protein expression in ICH model mice.

Among cerebrovascular events, ICH stands out due to its mortality and morbidity rates, often resulting in long‐term functional impairment.^3^ The pathophysiology of ICH involves a cascade of events beginning with hematoma formation, which subsequently leads to mechanical brain tissue damage as the hematoma expands, exerting a mass effect. Following the primary insult, secondary injury mechanisms perpetuate brain tissue damage and neuronal demise, fueled by perihemorrhagic inflammation, toxic blood breakdown products, and perihemorrhagic edema.^15^ Disruption of the BBB precipitates cerebral environmental homeostasis disturbances, culminating in cerebral edema.^16^ Traditionally, investigations into brain environmental homeostasis disruption have focused primarily on intrinsic brain alterations. However, recently increasing attention has been given to elucidating the intricate interplay between peripheral and CNS dynamics.^17^ Increases in proinflammatory cytokine levels may aggravate tissue injury, whereas increases in anti‐inflammatory cytokine levels might be protective in the brain after ICH.^2^ Inflammatory cytokines, such as IL‐6, which programs microglia to rebuild damaged brain vasculature, have been studied as therapeutic targets in a variety of acute and chronic brain diseases,^18^ while CCL5‐mediated astrocyte‐T‐cell interactions disrupt the BBB in mice after hemorrhagic stroke.^19^

In our study, we sought to elucidate the role of peripherally derived anti‐inflammatory factors in ameliorating BBB compromise and neurobehavioral impairment following cerebral hemorrhage, specifically by activating the IL‐10R/JAK1/STAT3/matrix metalloproteinase 9 (MMP9) signaling pathway in murine models. Our novel findings demonstrated that after ICH, there was an upregulation of IL‐10 expression within brain tissue, peaking around the third day after ICH. Conversely, peripheral IL‐10 levels decreased after ICH, reaching their lowest point around the third day after ICH, followed by a gradual increase. This temporal profile suggests that the endogenous anti‐inflammatory response is activated at the primary injury site in the brain, augmenting the expression of inflammatory mediators to inhibit the inflammatory cascade. Simultaneously, peripheral anti‐inflammatory factors use various routes to reach the injury site, where they exert their immunomodulatory effects.

IL‐10 is a pivotal anti‐inflammatory cytokine predominantly synthesized by microglia and astrocytes within the brain milieu, and its receptor, IL‐10 receptor alpha (IL‐10Ra), is ubiquitously expressed across various cell types.^20^ The cell receptors of IL‐10 belong to the type 2 cytokine family, which are transmembrane glycoproteins with 21 amino acids in the extracellular region, including two tandem type III fibronectin domains. Activated IL‐10R is a signal transduction complex containing two distinct receptor chains: IL‐10Ra and IL‐10Rβ. IL‐10Ra has a long amino acid chain and is the main signal transduction component. IL‐10R2β is a short transmembrane peptide chain with few components in the membrane. The IL‐10R complex is formed in two steps: first, IL‐10 binds to the high‐affinity receptor long chain IL‐10Ra; Second, IL‐10Rβ binds with low affinity. The complex consists of two IL‐10 dimers and four soluble forms of IL‐10Ra. The IL‐10/IL‐10R1a/IL‐10Rβ complex has biological activity and can activate JAK kinase and cause the phosphorylation of STAT factors, thus affecting mRNA transcription and regulating IL‐10.^21^, ^22^ Functionally, IL‐10 exerts diverse actions within the CNS, including the mitigation of neuroinflammatory processes, the augmentation of microglial phagocytic activity, the regulation of neuronal functionality, and the facilitation of neurogenesis.^4^, ^9^, ^20^, ^23^ In addition, in other pathological contexts, investigations have shown the ability of IL‐10 to safeguard retinal ganglion cells against growth factor‐induced apoptosis through the activation of STAT3 via phosphorylation.^24^, ^25^ The present study delineates the pivotal contribution of peripheral IL‐10 in ameliorating perihematomal edema by attenuating BBB breakdown, thereby improving the prognosis of ICH patients. These results reveal a new target that can be used for the treatment of cerebral hemorrhage and provide new ideas for the treatment of cerebral hemorrhage.

However, several inherent limitations warrant acknowledgment within the scope of this investigation. First, the widespread expression of IL‐10R across diverse cellular populations, coupled with the ubiquity of JAK1 and STAT3 as canonical inflammatory signaling transducers, renders the identification of the dominant cell types orchestrating the actions of IL‐10 challenging within the limits of this study. Furthermore, the inherent proteinaceous nature of IL‐10 makes it susceptible to enzymatic degradation within the body. The effective utilization of IL‐10 as a therapeutic agent necessitates the development of a suitable drug delivery mechanism to facilitate adequate IL‐10 deposition within the perihematomal region. Indeed, the intrinsic limitations associated with direct IL‐10 administration for the treatment of CNS pathology underscores the need for innovative strategies to optimize therapeutic efficacy.

## 5 CONCLUSIONS

Our investigation revealed that peripheral IL‐10 mitigates BBB compromise and neurobehavioral deficits after ICH, potentially through modulation of the IL‐10R/JAK1/STAT3 signaling axis. An IL‐10R‐knockout plasmid attenuated this effect, resulting in a reduction in IL‐10R expression. Consequently, IL‐10 has emerged as a promising therapeutic target for the clinical management of BBB disruption and neurological impairment resulting from cerebral hemorrhage.

## AUTHOR CONTRIBUTIONS

YC, QH, GZ, and HF designed the present study. YX, KW, YD, WY, XR, and WL were responsible for the data collection, statistical analysis and the data interpretation. YX, QH, and YC contributed to writing and editing the manuscript. All authors read and approved the final manuscript.

## CONFLICT OF INTEREST STATEMENT

The authors declare that they have no competing interests.

## Supporting information


Figure S1.

Table S1.

## Data Availability

All data generated or analyzed during this study are included in this published article and its supplementary information files.
