# Supplementary figures and images for: Peripheral cytokine interleukin‐10 alleviates perihematomal edema after intracerebral hemorrhage via interleukin‐10 receptor/JAK1/STAT3 signaling
Source: CNS Neurosci Ther. 2024 Jun 12;30(6):e14796. doi: 10.1111/cns.14796 (PMC11168964; doi:10.1111/cns.14796)

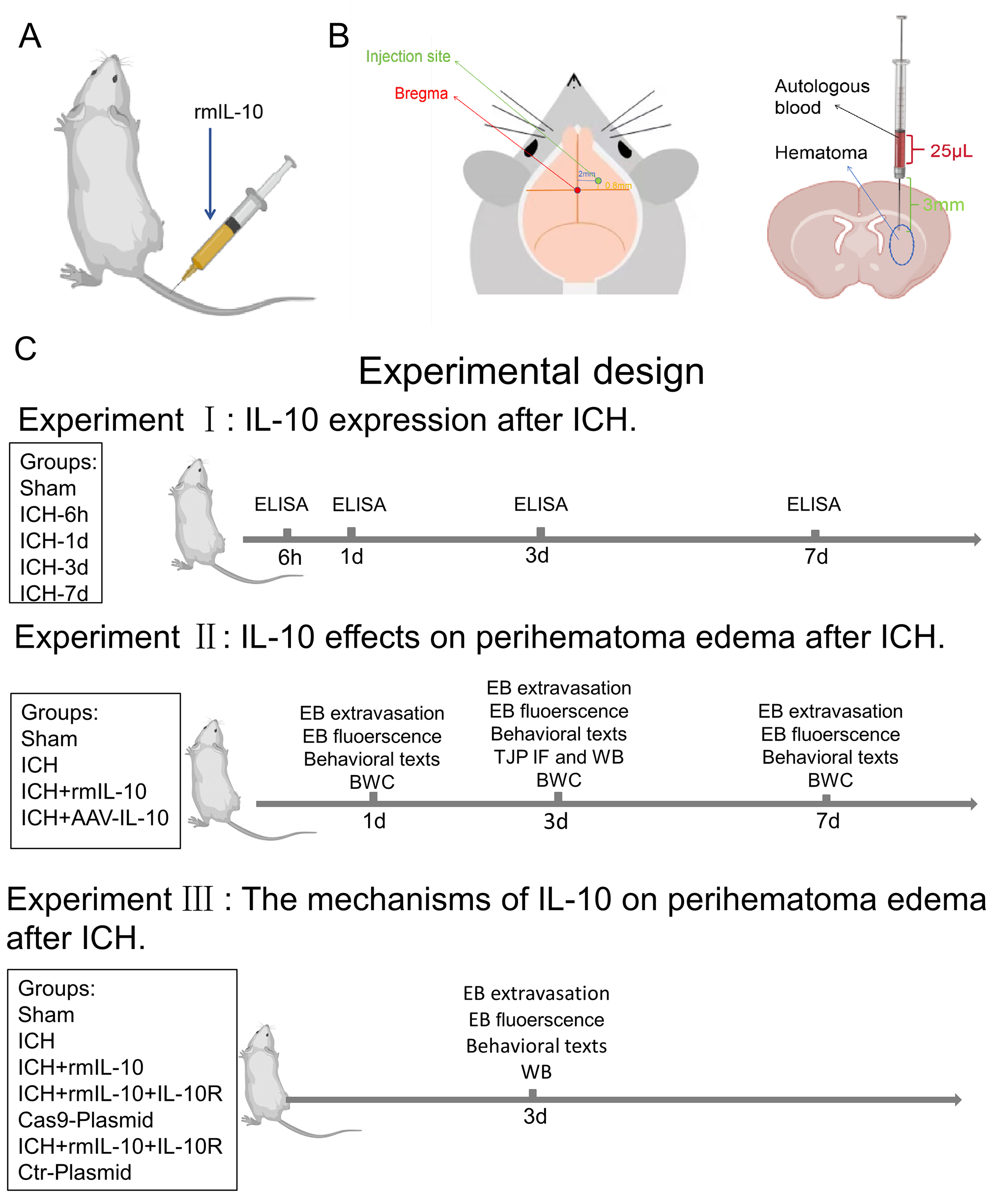

Supplement: Supplementary file 1 — Figure S1. [file CNS-30-e14796-s001.tif]
