# Supplementary material for: Peripheral cytokine interleukin‐10 alleviates perihematomal edema after intracerebral hemorrhage via interleukin‐10 receptor/JAK1/STAT3 signaling
Source: CNS Neurosci Ther. 2024 Jun 12;30(6):e14796. doi: 10.1111/cns.14796 (PMC11168964; doi:10.1111/cns.14796)
Supplement: Supplementary file 2 — Table S1. [file CNS-30-e14796-s002.docx]

**Supplemental Table 1.** Summary of experimental groups and mortality rate in the study.

| **Experimental Groups** |  | | | | |  | | **Mortality**  **(%)** | **Subtotal** |
| --- | --- | --- | --- | --- | --- | --- | --- | --- | --- |
|  |  | | | | | | **Exclusion** |  |  |
| **Experiment 1** | **ELISA** | | | | | |  |  |  |
| Sham | 6 | | | | | | 0 | 0 | 6 |
| ICH-6h | 6 | | | | | | 0 | 0 | 6 |
| ICH-1d | 6 | | | | | | 0 | 0 | 6 |
| ICH-3d | 6 | | | | | | 0 | 0 | 6 |
| ICH-7d | 6 | | | | | | 0 | 0 | 6 |
| **Experiment 2** | **Behavioral Tests (n=6)** | | | | | |  |  |  |
|  | **Brain water content** | **WB** | **IF** | **Evans Blue Etravasation** | **Evans Blue Fluorescence** | |  |  |  |
| Sham | 6 | 6 | 2 | 6 | 2 | | 0 | 0 | 22 |
| ICH (day 1, day 3, day 7) | 18 | 6 | 2 | 18 | 6 | | 0 | 0 | 50 |
| ICH+IL-10 (day 1, day 3, day 7) | 18 | 6 | 2 | 18 | 6 | | 0 | 2(3.85%) | 52 |
| ICH+IL-10- (day 1, day 3, day 7) | 18 | 6 | 2 | 18 | 6 | | 0 | 4(7.41%) | 54 |
| **Experiment 3** |  |  |  |  |  | |  |  |  |
| Sham | - | - | - | - | - | | - | - | - |
| ICH | - | - | - | - | - | | - | - | - |
| ICH+IL-10 | - | - | - | - | - | | - | - | - |
| ICH+IL-10+IL-10R Plasmid | - | 6 | - | 6 | 2 | | 0 | 2(12.5%） | 16 |
| ICH+IL-10+Ctr Plasmid | - | 6 | - | 6 | 2 | | 0 | 0 | 14 |
| **Total** | 60 | 36 | 8 | 72 | 24 | | 0 | 8(3.36%) | 238 |

ICH, intracerebral hemorrhage; WB, western blot; IF, immunofluorescence; Ctr, control. “-” means the mice/samples required in this group can be shared with other existing groups.
